# Pathological complete response induced by first-line chemotherapy with single agent docetaxel in a patient with advanced non small cell lung cancer

**DOI:** 10.1186/1477-7819-8-8

**Published:** 2010-02-05

**Authors:** Ferdinando Riccardi, Giuseppe Di Lorenzo, Carlo Buonerba, Guglielmo Monaco, Roberto Monaco, Mimma Rizzo, Sarah Scagliarini, Florinda Scognamiglio, Marilena Di Napoli, Giacomo Carteni'

**Affiliations:** 1 UOC Oncologia, Ospedale Cardarelli, Napoli, Italy; 2 UOC Chirurgia Toracica, Ospedale Cardarelli, Napoli, Italy; 3 UOC Anatomia Patologica, Ospedale Cardarelli, Napoli, Italy

## Abstract

**Background:**

Defining the optimal treatment for patients with inoperable non small cell lung cancer (NSCLC), presenting with metastatic mediastinal lymph nodes, is challenging. Nevertheless, preoperative chemotherapy or radiotherapy might offer a chance for these patients for radical surgical resection and, possibly, complete recovery.

**Case Presentation:**

A 62-year old man with IIIA-N2 inoperable NSCLC was treated with first-line single agent docetaxel. A platinum-based treatment, though considered more active, was ruled out because of renal impairment. The patient tolerated the treatment very well and, although his initial response was not impressive, after 14 cycles he obtained a complete clinical response, which was confirmed pathologically after he underwent surgical lobectomy.

**Conclusion:**

In non-operable NSCLC patients not eligible for a platinum-based treatment, single-agent docetaxel can provide complete pathologic responses. Failure to obtain a response after the first few cycles should not automatically discourage to continue treatment.

## Introduction

In non-small cell lung cancer (NSCLC), mediastinal lymphnode involvement comprises a wide spectrum of severity. In fact, while 9% of patients with IIIA-N2 NSCLC is candidate for surgery at diagnosis, with a 5-year survival of 20-30%, the majority of patients with stage IIIA-N2 and IIIB-N2 tumors cannot undergo up-front surgical resection and present a 5-year survival rate of about 5%[1]. There is a growing body of evidence that suggests that neoadjuvant chemotherapy or radiotherapy or chemoradiotherapy prior to surgery can be advantageously employed in patients with stage IIIA-N2 disease[2]. We herein describe an unusual case of pathological complete response induced by single agent docetaxel chemotherapy in a patient with diagnosis of NSCLC initially judged not fit for surgical resection.

## Case Presentation

**Figure 1:**
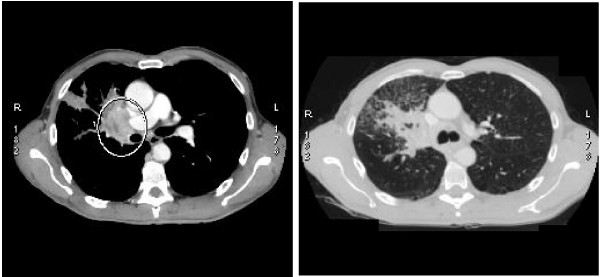
**CT scan on June 2008**.

A 62-year-old man was referred to Cardarelli Hospital, Naples, in June 2008. He presented a 4-month history of non productive cough, progressive shortness of breath, increasing abdominal girth and anorexia. He was mildly dyspnoeic at rest and complained about severe asthenia. There was no alcohol abuse in the previous ten years, while he presented an 80-pack-a year history of cigarette use, as he had smoked since the age of 18 until recently. An abnormal shadow appeared on the patient's chest X-ray, while a CT scan revealed a right hilar tumor, measuring 92 × 66 mm, as shown in Figure 1.

A fiberoptic bronchoscopy indicated the presence of an endobronchial lesion infiltrating the upper lobar bronchus of the right lung. Pathological analysis with CT-guided fine-needle aspiration biopsy (FNAB) of the collected specimens showed that the tumor was a poorly differentiated squamous cell carcinoma. There was no evidence of extra thoracic metastasis on brain MRI, abdominal CT and FDG-PET scans. Therefore, the patient had a stage IIIA-N2 NSCLC.

**Figure 2:**
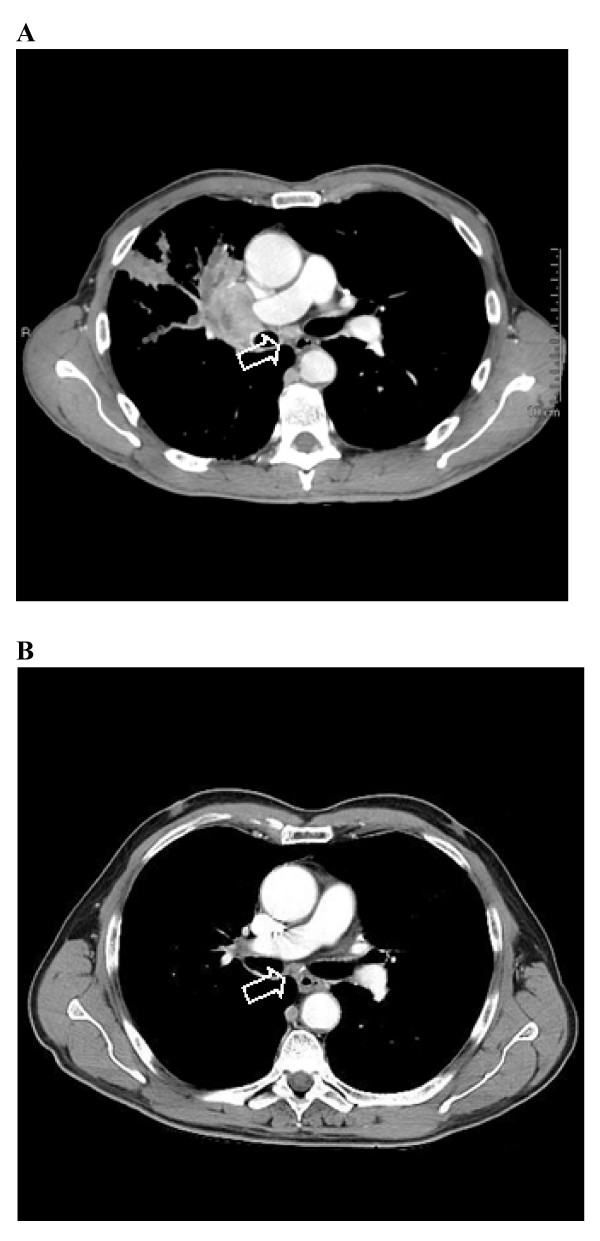
**Chest computed tomography scan (A) before treatment of docetaxel (B) 10 months after treatment**. The arrows show the mediastinal lymph node metastasis that gradually reduced in size.

**Figure 3:**
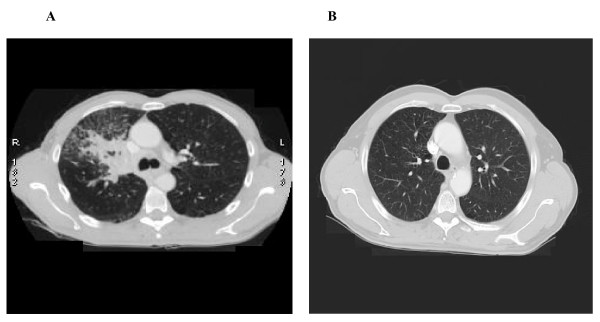
**(A) CT scan June 2008 before therapy**. (B) CT scan April 2009 after therapy.

Blood tests revealed a mild renal failure with an estimated creatinine clearance of 47 ml/min. Considering the impaired renal function, a platinum-based treatment was ruled out. Therefore, the patient was started on single-agent docetaxel induction chemotherapy (75 mg/sqm over 60 min on day 1, every 3 weeks). The first evaluation was made after four cycles. In September 2008, a CT scan showed a partial response of the carcinoma, which appeared to measure 65 × 41 mm. The safety profile was good, with patient reporting grade 1 anemia, grade 3 neutropenia without fever, weight loss inferior to 10% and asthenia. Therefore, the treatment was continued for four additional cycles. In January 2009, a CT scan showed an impressive shrinkage of the hilar mass, measuring 35 × 30 mm. There also was a marked clinical improvement, as the cough and the dyspnoea had disappeared and the patient reported a general feeling of wellness. Considering the patient's good compliance, it was decided to continue the same treatment until progression or unacceptable toxicity. After 14 cycles, both CT and PET scans showed further tumor response, as the right hilar mass measured only 16 × 10 mm, and the patient was finally judged to be eligible for surgery (figures 2 and 3). The chest surgeon decided to perform a right lobectomy with mediastinal lymph node sampling. On pathological examination, the response to induction chemotherapy was complete.

## Discussion

Preoperative, platinum-based combination chemotherapy is safe and active in NSCLC, although a randomized trial reported a modest, not statistically significant excess of post-surgical morbidity and mortality[3]. As single-agent therapy is associated to a lower response rate, but also to lower toxicity[4], single-agent docetaxel chemotherapy might be feasible for preoperative therapy, especially in the context of an early stage disease to be cured with surgery and adjuvant therapy, as well. A phase III trial randomized patients with stage IIIA or locally treatable IIIB NSCLC to neoadjuvant docetaxel (up to three 3-weekly cycles at 100 mg/m^2^, 134 patients) or no chemotherapy (140 patients) prior to surgery/curative-intention radiotherapy. Treatment proved to be safe and well tolerated, but this trial showed only a trend in survival prolongation (14.8 months in the chemotherapy group vs. 12.6 months in the control group, not statistically significant)[5]. A multicenter phase III trial indicated that patients with advanced NSCLC receiving the combination of docetaxel and cisplatin (DC) had a statistically significant improvement in objective response rate compared with patients treated with docetaxel alone (D), although there was no statistically significant difference in overall survival (36% vs. 18% and 10.5 vs. 8.0 months, respectively). Furthermore, patients treated with single-agent docetaxel had a more favorable toxicity profile than patients treated with DC. In particular, there were important differences in the incidence of: (a) grade 2 anemia, (b) grade 3/4 nausea/vomiting, diarrhea, and neurotoxicity, (c) nephrotoxicity of any grade and (d) treatment-related deaths (5 in the DC arm vs 1 in the D arm)[6].

Similarly, the toxicity profile of the monotherapy arm in the Lilenbaum [7] study was more favorable compared with that of the combination arm, with no difference in survival.

Although it is generally believed that best responses are obtained in patients who initially respond to docetaxel treatment, the case presented herein proves how an unexpected, striking complete pathologic response can be achieved with a long-term treatment, which was substantially well tolerated. Such an observation, if confirmed by large-sample studies, might indicate how an unsatisfactory initial response, but not progressive disease, does not constitute a reliable predictive factor for response, as a great improvement in response itself can still be obtained, if treatment is continued. Noteworthy, such a result appears to be in contrast with observations regarding neo-adjuvant docetaxel-based chemotherapy in other inoperable malignant tumors, such as breast cancer[8].

Surprisingly, a complete pathologic response was not obtained with a platinum-based regimen, but with single-agent docetaxel, which is not considered to be the first-line chemotherapy for non-operable patients with NSCLC. The search for either molecular or genetic markers predictive for docetaxel sensitivity might constitute future targets of investigation.

Finally, we cannot but underline our decision not to suspend treatment as soon as the patient was considered to be eligible for surgery. In fact, our choice to continue docetaxel chemotherapy resulted in further tumor shrinkage, with no additional toxicity, and gave the patient the possibility to undergo a lobectomy rather than a pneumectomy, with the obvious advantages of a less invasive and more limited surgical operation. Our patient obtained a striking response to docetaxel as first-line monotherapy treatment and could undergo an upper right lobectomy with radical intent. Microscopic examination showed a pathological complete response of the neoplasia.

## Conclusion

Our results suggest that physicians should be aware of potential objective responses to Docetaxel, as first-line, for patients with limitations to receive platinum-based regimens, even after the tumor does not seem to be reduced in size at the beginning phase of the treatment.

## Consent

Written informed consent was obtained from the patient for publication of this case report and accompanying images. A copy of the written consent is available for review by the Editor-in-Chief of this journal.

## Conflict of interests

The authors declare that they have no competing interests.

## Authors' contributions

FR, GDL and GC conceived of the study, coordination and writing manuscript;

GM diagnosed the cancer with fiberoptic bronchoscopy and decided to perform a right lobectomy; RM: Pathologist and author of diagnosis of poorly differentiated squamous cell carcinoma;CM, MR, SS, FS, MDN esamined the patient at follow-up.
